# Satisfaction can co-exist with hesitation: qualitative analysis of acceptability of telemedicine among multi-lingual patients in a safety-net healthcare system during the COVID-19 pandemic

**DOI:** 10.1186/s12913-022-07547-9

**Published:** 2022-02-14

**Authors:** Michelle-Linh T. Nguyen, Faviola Garcia, Jennifer Juarez, Billy Zeng, Elaine C. Khoong, Malini A. Nijagal, Urmimala Sarkar, George Su, Courtney R. Lyles

**Affiliations:** 1 Medicine, Division of General Internal Medicine, University of California San Francisco, 1001 Potrero Avenue, San Francisco, CA 94110 USA; 2 Center for Vulnerable Populations, Zuckerberg San Francisco General Hospital and Trauma Center, San Francisco, CA USA; 3 Obstetrics & Gynecology, University of California San Francisco, San Francisco, CA USA; 4 Medicine, Division of Pulmonary and Critical Care, University of California San Francisco, San Francisco, CA USA

**Keywords:** Telemedicine, Multilingual, Safety-net, Telehealth, Acceptability

## Abstract

**Background:**

The COVID-19 pandemic triggered unprecedented expansion of outpatient telemedicine in the United States in all types of health systems, including safety-net health systems. These systems generally serve low-income, racially/ethnically/linguistically diverse patients, many of whom face barriers to digital health access. These patients’ perspectives are vital to inform ongoing, equitable implementation efforts.

**Methods:**

Twenty-five semi-structured interviews exploring a theoretical framework of technology acceptability were conducted from March through July 2020. Participants had preferred languages of English, Spanish, or Cantonese and were recruited from three clinics (general medicine, obstetrics, and pulmonary) within the San Francisco Health Network. Both deductive and inductive coding were performed. In a secondary analysis, qualitative data were merged with survey data to relate perspectives to demographic factors and technology access/use.

**Results:**

Participants were diverse with respect to language (52% non-English-speaking), age (range 23-71), race/ethnicity (24% Asian, 20% Black, 44% Hispanic/Latinx, 12% White), & smartphone use (80% daily, 20% weekly or less). All but 2 had a recent telemedicine visit (83% telephone). Qualitative results revealed that most participants felt telemedicine visits fulfilled their medical needs, were convenient, and were satisfied with their telemedicine care. However, most still preferred in-person visits, expressing concern that tele-visits relied on patients’ abilities to access telemedicine, as well as monitor and manage their own health without in-person physical evaluation.

**Conclusions:**

High satisfaction with telemedicine can co-exist with patient-expressed hesitations surrounding the perceived effectiveness, self-efficacy, and digital access barriers associated with a new model of care. More research is needed to guide how healthcare systems and clinicians make decisions and communicate about visit modalities to support high-quality care that responds to patients’ needs and circumstances.

**Supplementary Information:**

The online version contains supplementary material available at 10.1186/s12913-022-07547-9.

## Background

The Coronavirus Disease 2019 (COVID-19) pandemic triggered unprecedented expansion of outpatient telemedicine encounters throughout all medical settings in the United States (U.S.) [1, 2]. In contrast to before the pandemic, when telemedicine was being used primarily in specialized patient populations (e.g. heart failure) or to increase access to care for rural patients, in August 2020, telemedicine encounters (including telephone and video visits) accounted for 50-60% of all primary care delivered in two large delivery systems in San Francisco—including the study site for this research [3]. Given this widespread experience with and normalization of telemedicine in all settings in the United States, almost all healthcare settings are preparing to continue telemedicine into the future [4–7].

Telemedicine is defined by the U.S. Health Resources and Services Administration as “ … the use of telecommunications and information technologies to support long distance clinical health care...” [8] For the purposes of this paper, we use “telemedicine” to refer to scheduled telephone and video encounters for outpatient clinical care. Although most U.S. health systems have strived to provide both telephone and video encounters, different delivery systems and patient populations have experienced the transition to telemedicine encounters differently. Specifically, safety-net healthcare delivery settings have relied almost exclusively on telephone visits [9], while medical centers serving primarily privately-insured and/or higher-income patients have had higher uptake of video visits [10]. This difference has potential to increase structural and individual health inequity, especially given that in the United States, insurance payments for telephone visits will likely be lower than video visits following the pandemic and therefore may no longer be provided as an option [11]. While there are clear organizational and infrastructure differences between the safety-net and other medical settings that influence these patterns, visit modality is likely also influenced by historical, social and individual determinants. For example, a safety-net healthcare delivery system may offer video visits, but uptake will still be limited if the patients they serve face barriers to digital device/connectivity access [12–15], consider telemedicine visits inferior in quality to in-person visits, and/or have higher trust or preference for in-person visits and communication with providers [16].

Although two recent studies have demonstrated that many safety-net patients are open and interested in telemedicine visits, specifically video visits [12, 17], there is a need to contextualize and add depth to these findings to ensure that ongoing implementation efforts are sensitive to patients’ needs and context. It is especially vital that we highlight the experiences and perspectives of patients with lower incomes and who face structural barriers to technology use as to avoid designing and building systems that increase inequity. In order to fill this need, we conducted semi-structured interviews with safety-net patients sampled for a wide range of ages, language preferences, technology access, and clinical settings.

## Methods

### Study design/setting

We performed 25 semi-structured interviews between March and July 2020. We used a theoretical framework of acceptability to guide interviews and explore whether participants’ considered telemedicine (including telephone and video) visits appropriate [18]. This framework guided us in exploring domains of acceptability, such as: affective attitude (how an individual feels about an intervention), burden (the perceived amount of effort that is required to participate in the intervention), perceived effectiveness (the extent to which the intervention is perceived as likely to achieve its purpose), and self-efficacy (the participant’s confidence that they can perform the behavior(s) required to participant in the intervention) [18].

In order to obtain a range of experiences, English-, Spanish-, and Cantonese-speaking patients who were scheduled for telemedicine visits were purposively sampled from the patient panels of three clinics—general medicine, obstetrics, and pulmonary--within the San Francisco Health Network, which is the public healthcare delivery system in the city and county of San Francisco, California, serving almost exclusively Medicaid and other uninsured/publicly-insured patients. We chose these three languages because together they account for the preferred language of 90% of patients receiving care in this health system. 105 qualifying participants were identified, 63 participants were contacted, and 25 participated in the study.

### Study procedures

Participants were interviewed by bilingual study staff (including BZ, EK, FG) in their preferred language over the telephone. All interviews were recorded. Interviews included discussion of participants’ experiences with recent telemedicine visits, as described above. We asked the two participants who had not experienced a telephone or video visit about their openness to participate in this type of visit and their anticipated preferences. Participants were reimbursed for their time up to $40. We used a combination of bilingual study staff (BZ, FG) and professional translation services to translate and transcribe transcripts into English.

For our quantitative data collection, trained research assistants (two of whom were bilingual) conducted surveys with all participants before their interviews. Surveys included demographic information (age, preferred language, gender, self-identified race and ethnicity, education level, health literacy), as well as their access to and use of digital devices, the Internet, and smartphone applications (including video chat applications). Participants from the primary care and pulmonary clinics, all of whom had experienced either a telephone or video visit in the two weeks preceding their interview, were also asked to rate their visit and answer whether all of their medical needs were met.

### Analysis

First, we summarized the quantitative survey data among all participants. Next, we conducted a thematic analysis using deductive coding according to the theoretical framework that guided the semi-structured interviews [18], combined with inductive coding to allow for novel ideas to emerge. Dedoose software was used for coding. We underwent a group consensus process to ensure intercoder consistency. More specifically, the same interview transcripts were coded by all members of the coding team (CRL, FG, JJ, BZ), after which we held multiple group meetings to discuss and review differing coding applications until consensus was achieved. MTN, CRL, and FG independently looked for and jointly discussed common themes across all transcripts. All survey items were descriptively summarized using Excel software. In a secondary analysis, we used the quantitative survey data to supplement our qualitative results by reviewing excerpts stratified by clinic, preferred language, gender, age categories (20-49 years old vs. 51-80 years old), health literacy (high health literacy vs. all others), and Internet use (several times per day vs. once per day or less).

## Results

Participants represented a broad range of spoken language, age (range 23-71 years), self-identified race/ethnicity, & technology use (Table 1).

**Table 1:** Demographic and technology access/use characteristics

**Characteristics (*N*** **= 25)**	
Age in years, median (range)	42 (23-71)
Interview language, n (%)	
English	12 (48)
Spanish	8 (32)
Cantonese	5 (20)
Gender, n (%)	
Female	17 (68)
Race or Ethnicity, n (%)	
Asian or Pacific Islander, or other	6 (24)
Black	5 (20)
Hispanic/ Latino (a)	11 (44)
White	3 (12)
Education, n (%)	
Less than high school	4 (16)
High school graduate/ GED	5 (20)
Some college	9 (36)
College graduate or more	7 (28)
Appointment type in last two weeks, *n* (%)	
None	2 (8)
Video	4 (16)
Phone	19 (76)
**Technology Access and Use**	
Do you have Internet service at home other than via your smartphone (such as WiFi)?, *n* (%)	
Yes	20 (80)
How often do you use the Internet? (on your smartphone, using a computer or tablet device), n (%)	
Several times a day	16 (64)
About once a day	4 (16)
About once a week	2 (8)
Less than once a week	1 (4)
I do not use the internet	2 (8)
How often do you use apps (for any purpose) on your smartphone?, n (%)	
At least once a day	20 (80)
Once or several times a week	2 (8)
Once or several times a month	2 (8)
Never	1 (4)
Do you use video chat applications? (WhatsApp, Messenger, FaceTime, Google Hangout, Zoom), n (%)	
Yes	20 (80)

All but two had experienced a recent telemedicine visit with a clinician. Out of the 23 telemedicine visits, 19 were telephone visits and 4 were video visits.

### Overarching themes

**Fig. 1:**
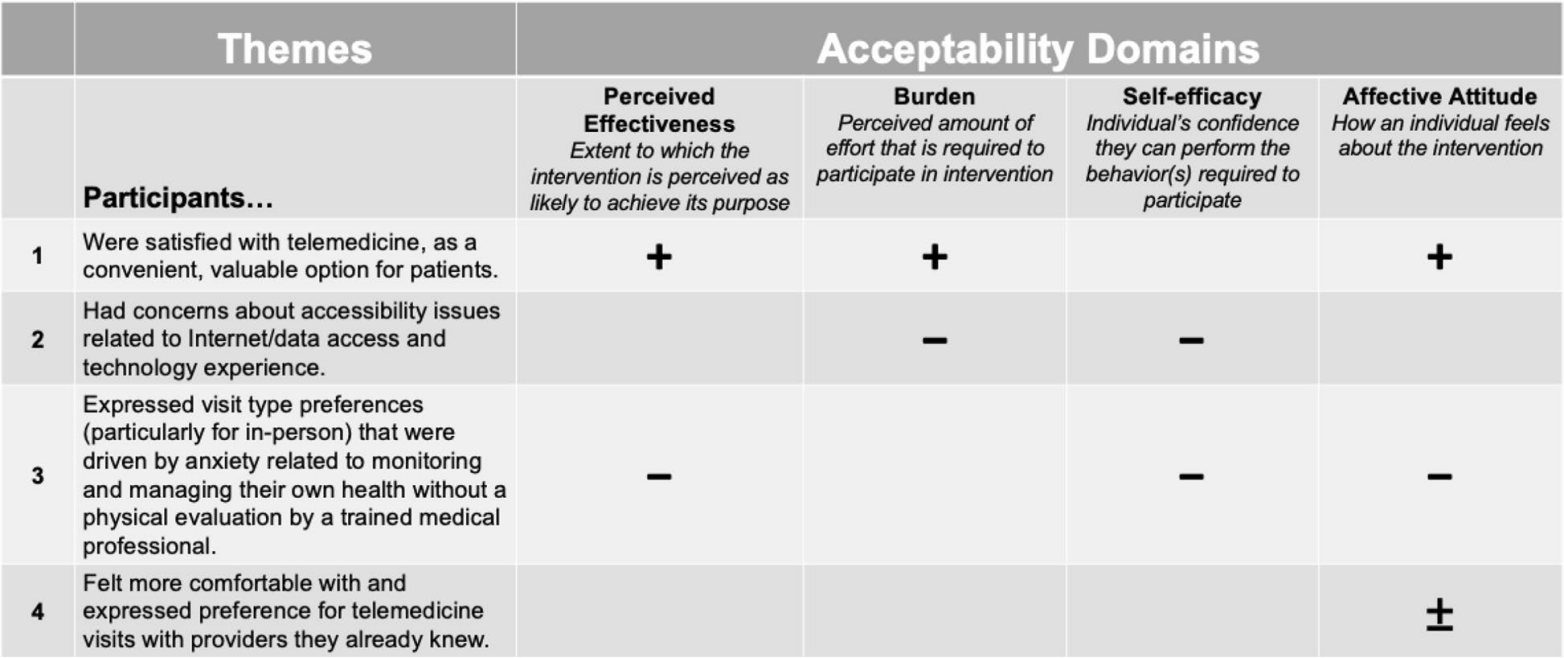
Qualitative themes mapped to domains of telemedicine acceptability. Plus (+), minus (–), and neutral signs (±) represent positive, negative, and neutral drivers of telemedicine acceptability, respectively

Our findings centered around four major, cross-cutting themes, which mapped to the constructs of affective attitude, burden, perceived effectiveness, and self-efficacy from the acceptability framework used in our coding (Fig. 1). While we also reviewed coded excerpts stratified by patients’ preferred language, gender, age, health literacy, and Internet access/use, we found no substantial differences between these groups.

#### Theme 1: participants were satisfied with telemedicine as a convenient, valuable option for patients

Almost all participants felt satisfied with their telemedicine care and felt their needs were fulfilled. Participants were accepting of telephone and video visits as comfortable, convenient, and valuable options for patients. For example, the majority of participants expressed positivity about saving travel time and not having to wait for visits. They also recognized that having the option for telemedicine would make accessing medical care easier for people who work, take care of children, or have limited mobility. Representative quotes are summarized in Table 2.

**Table 2:** Representative quotes for Themes 1 & 2 related to satisfaction, convenience, value, and accessibility

Theme	Quotes
Telemedicine visits were satisfactory.	*“The doctor was very thorough online. She wasn’t just asking me like, ‘Physically, how are you feeling? Do you have these symptoms? Do you have any bleeding? Do you have any of these?’ She also asked me, ‘Emotionally, how are you feeling? Mentally?’ and stuff like that. … I think that was what she can do online, where you can only do a verbal assessment, then I think it went well.”* *“ … prior to having these visits I was suspicious. Like in my mind I didn’t know if the phone call was good enough. But after having this experience, I felt it was pretty good, there didn’t seem to be any difference at all.”*
Telemedicine visits were more convenient than in-person visits.	*“It’s more convenient because I don’t have to wait. And traveling to the SFGH, transferring buses, takes so long, waiting for like 30-45 minutes.”* *“I don’t always have time to go to the hospital, but I can receive a visit call during my lunch break at work.”* *“Some people are busy with their children and can’t go out, or they don’t have time to go to their appointments. They won’t miss their appointments. They still get them. I think that’s good. It’s a good option.”*
Telemedicine visits were considered a valuable option for patients.	*“I know it’s a big deal to put a whole new system in place … , but I would say it would be beneficial. Some people really can’t get out without having the assistance of an ambulance or something.”* *“It’s going to be interesting and a good one because sometimes … in this kind of … situation, pandemic, lockdown … it’s going to help us to still keep in touch with our doctor, keep in touch with our nutritionist, … keep in touch with our health condition.”* *“Well, sometimes you make the trip to the doctor not very happily because the attention isn’t very good. … They are practically without contact. There are visits that don’t require examining the patient. I think those would be better [via telemedicine].”*
Patients had concerns about accessibility.	*“ … the problem we have now that my child is in school and with his classes and we had little Internet. And we all had telephones, but not much coverage for Internet.”* *“ … not all people it’s going to be easy to understand. For the millennial people like the younger people, it maybe is going to be easy … but older people, it’s going to be hard so maybe guide … the patient first ...”* *“They got to sit me down and show me. I mean, I’ve never done that. … I haven’t done that … because now I’m old.”*

#### Theme 2: participants had concerns about accessibility issues related to Internet/data access and technology experience

Patients were aware of their own accessibility barriers, such as limited access to Internet and cellular data, as well as potential access barriers for certain groups of patients such as older people (Table 2).

#### Theme 3: participants expressed visit type preferences (particularly for in-person visits) that were driven by anxiety related to monitoring and managing their own health without undergoing a physical evaluation by a trained medical professional

Despite their acceptance of telemedicine visits, participants generally favored a) in-person care more than video visits (Table 3) and b) video visits more than telephone visits. Despite most not having experience with video visits, many participants felt that video visits would be better than telephone visits because it would more closely resemble an in-person interaction in which the doctor could see them.

**Table 3:** Representative quotes for Theme 3, related to visit modality preference

Theme	Quotes
In-person preference	*“I would always prefer in person visit just because all of my vitals get taken … etcetera, etcetera. I have a number of issues. So, the preference would always be that, but I don’t have any problem doing it the other way [telemedicine] unless there is something that has to be in person.”* *“Yes, in both, the doctor has visual contact with you, but there can’t be much examination in the video visit. You can just show and he can see. It’s better than not seeing anything and not being able to communicate, but the [in-person] visit offers much more benefit.”* *“Yes, that can be handled by video or telephone but I feel to an extent that I’m giving up something beneficial for my health by not seeing the providers in person.”* *“Honestly, in-person quality of care was better just because they’re actually able to perform all the essential features of a visit that they’re not able to give you over a video.”*
Video visit preference over telephone visit	*“As I look at video visit, I think it would still be a lot better than over the phone just because … you would be able to show the doctor like where it hurts. If you have a rash or something, just show it to them...”* *“The video one was a little bit better than the phone one just because I was actually able to see them better, I was … able to point out things, they were … able to swipe over the video to see the concerns that I was having...”* *“I think a video visit would be better so the doctor could see me. She could see how I take my blood pressure and I could show her my record that I have here. She could see it herself. She could tell if I’m doing things correctly and not deceiving her.”* *“I feel that the doctor would at least be seeing me. He couldn’t touch me, but he could see me. I don’t know. It would be different.”*
Anxiety and lack of confidence in self-monitoring and self-managing health without in-person physical evaluation by a trained medical professional	*“It’s different to have a doctor check you and assess you based on what’s physically going on with you and hearing these things and seeing it himself than for you to try to explain. I feel like, as patients, unless we’re trained as medical physicians, we don’t have the words sometimes to explain...”* *“It’s a different feeling as a person that has a disease. … they’ve told me that I’m getting four stages … I’m in stage III. That means that’s the end of it. … I think going in, when I get my blood pressure taken by the doctor, I get a blast of relief that lasts me till the next visit. … I’m old. I like to see them. I’m not digging the phone visits.”* *“ … over the phone, they would ask like, “Do you feel the baby moving? How do you feel? How is everything going?” but there was no actual physical examination, so how is the doctor actually gauging that everything is actually okay, because I am not a doctor myself. I just answer the questions [to] the best of my ability … but there’s no actual real medical examination or procedure happening for the doctor to confidently tell you that everything’s okay through the phone.”* *“Well, you don’t get your weight checked, you don’t get your pulse checked, your blood pressure checked. I think that’s important for an old person every seven days … once a month, once every two months. You need your blood pressure checked by a professional, and temperature checked by a professional. … I’d like to know and have it charted … I’m speaking for the old aged people. I’m 71. Old people need to be actually on the scale and told to make them feel better.”*

In addition, the preference for in-person visits was driven by anxiety and lack of confidence in monitoring and managing their own health without physical evaluation by a trained medical professional (Table 3). Participants expressed that in-person visits provided a sense of reassurance that telemedicine visits did not. Part of this reassurance stemmed from physical evaluation with vital sign measurements and/or physical examination by a trained medical professional. Most participants felt that in-person visits were particularly more appropriate than telemedicine for evaluating severe pain:

*“What I’m thinking is like if it’s just a concern about the question or understanding something about the question, maybe still okay with the [telemedicine] visit, but if it’s something that you’re really in pain that you want show to your doctor, my suggestion is it’s much better if you can go to the visit face-to-face instead of the video call.”*

*“If it’s like a 4/10 pain then maybe it’s not needed to go in person. But if anything’s more severe or I really want the doctor to look at it I will still need to go.”*

Obstetrics patients, in particular, more often expressed anxiety regarding not being physically evaluated:

*“Yes, [I] was left with a feeling like, ‘Okay. She says everything’s okay. I answered to the best of my ability. I just hope everything’s okay,’ because that’s the only way I could ease my own mind, is to just hope that everything’s okay after that.”*

#### Theme 4: participants felt more comfortable with and expressed preference for telemedicine visits with providers they already knew

Finally, across the interviews, most participants expressed more reassurance and comfort with telemedicine if the visit was with a doctor or other clinician they already had a relationship with.

*“it’s better if I see them in person first if I never met them. Because I don’t know them that well. But for someone who I seen for a long time like my personal doctor I don’t need to go in person … For new doctors I haven’t seen before my first choice would be in-person then second video, lastly would be phone.”*

*“I’m glad I didn’t have to talk to anyone else. … That was my own personal doctor because I asked her about that first before she said about the Zoom and I was making sure like, “Is it going to be with you” and she said, ‘It would be me.’”*

*“I want to visit the doctor, the doctor I know already. If it is a new doctor, I prefer to see regular new doctor [in person]. Next time, the same doctor. I don’t want new doctors every video, every phone, and – yes, I want it to be like make sure the patient is comfortable.”*

## Discussion

To our knowledge, our study was the first to examine in-depth qualitative perceptions of acceptability of telemedicine among multi-lingual safety-net patients during the COVID-19 pandemic. Few telemedicine-focused studies have purposefully sampled participants speaking multiple languages, which is critical given the needs of healthcare systems to deliver telemedicine care seamlessly to patients who face structural barriers to healthcare.

We found that participants had high satisfaction with their current telemedicine encounters (which were primarily telephone visits) but generally preferred in-person visits and the associated medical support and physical examination. Our qualitative coding allowed a deeper exploration of patient perceptions and mapped themes to an existing theory. Our analysis provides a more nuanced understanding of telemedicine acceptability: how high satisfaction with telemedicine can co-exist with patient-expressed hesitations about perceived effectiveness, self-efficacy, and digital access barriers.

Our findings align with previous research that found high rates of satisfaction with telemedicine in other settings [17, 19, 20], as well as preferences for in-person relationships with clinicians [16]. They build upon prior literature that has demonstrated high initial interest in and perceived effectiveness of video visits and other digital healthcare modalities among racially/ethnically and/or linguistically diverse patients, including Spanish-speaking patients from our same healthcare delivery system [12, 21, 22].

Moving forward, research is needed to guide how healthcare systems and clinicians make decisions and communicate about visit modality with the overarching goal of utilizing multiple visit modalities to support personalized, high-quality care that responds to patients’ needs and circumstances. This includes understanding not only how to best utilize telemedicine versus in-person visits, but also how to best utilize telephone versus video visits. We need to better understand how to tailor our use of visit modalities and communicate this new standard of care to patients, making explicit that virtual care is not a “substitute” for in-person care but rather a valuable care option for patients who want more access to visits to supplement ongoing in-person relationships. This is especially important while caring for communities that have experienced exploitation and discrimination. Beyond clear communication to patients about when and why telemedicine might make sense for specific encounters, we also need to understand and uphold patient preferences for care. Many of our participants expressed the desire to have telemedicine visits with a clinician they already had an established relationship with. This should motivate health systems to prioritize continuity in their telemedicine implementation efforts. Overall, it is especially important to recognize that each patient has unique needs and some may need more time to consider how new care modalities help facilitate their health and wellness. Finally, we need to support digital access and training for patients, given our findings that patients may prefer video visits over telephone visits and yet there are known barriers to devices, data/Internet, and skills that impede interested patients from using digital modalities of care [14, 23].

An important limiting factor to our work is that interviews were conducted during the COVID-19 pandemic, which likely influenced how participants felt about and experienced telemedicine while also broadening almost all participants’ experiences trying out telemedicine for the first time. Several participants expressed relief at having telemedicine as an option for safety reasons, and it is unclear how their perspectives may change once the pandemic ends. Other limitations of this work include a single delivery system and small sample size within each clinic type. It is also unclear whether patients had any other experiences with telemedicine in other healthcare settings. Future studies that sample more video visit experiences are still needed to directly compare experiences with telephone visits versus video visits. Finally, our secondary analysis examining excerpted codes by patient factors, such as language and digital skills/experience, was exploratory in nature and should be repeated in future work engaging diverse patient populations in other settings.

## Conclusions

The widespread expansion of telemedicine has and will continue to be experienced differently by different patient populations. The option of telemedicine visits has the potential for decreasing access barriers for patients who work, are caretakers, have limited mobility, or limited access to transportation. This potential may not be realized if implementation is not sensitive to patient perspectives and needs. Telemedicine implementation efforts in the safety-net should prioritize clear two-way communication regarding visit modality selection that will not only fulfill clinicians’ standards for appropriate care but will also ensure that patients feel reassured and cared for in a high-quality and safe manner.

After the COVID-19 pandemic has ended, we must continue to support patients’ needs and preferences, recognizing that interest in, openness to, and satisfaction with telemedicine can co-exist with concerns and hesitations surrounding a new model of care.

## Supplementary Information

**Additional file 1. ** Interview Guide.

## Data Availability

The datasets generated during and/or analysed during the current study are not publicly available due to participants’ privacy concerns but are available from the corresponding author on reasonable request.

## Abbreviation

COVID-19: Coronavirus Disease 2019
