# Supplementary material for: Satisfaction can co-exist with hesitation: qualitative analysis of acceptability of telemedicine among multi-lingual patients in a safety-net healthcare system during the COVID-19 pandemic
Source: BMC Health Serv Res. 2022 Feb 14;22:195. doi: 10.1186/s12913-022-07547-9 (PMC8842908; doi:10.1186/s12913-022-07547-9)
Supplement: Supplementary file 1 — Additional file 1. Interview Guide. [file 12913_2022_7547_MOESM1_ESM.docx]

**Appendix: Interview Guide**

Walk us through how your recent visit went, from scheduling it to completing the visit with your provider.

- How was it scheduled and set up?
- Was it with a provider that you already knew, or a provider who you had never met in-person before?
- Any emotions or feelings about this type of visit?
- What are the next steps after this visit for your care?

What were the best parts about the visit?

What could have been improved or made the experience better?

How did you feel about the quality of the visit?

- Communication with doctor
- Answering all of your questions

How successful did you think you were at calling/signing into the visit on a computer or phone? Why?

(IF PHONE) Did someone in clinic offer to schedule this visit using video in addition to talking on the phone? If so, what did you think about using the video feature on your smartphone or computer?

What kind of help or support might/did you need to complete a video visit? Who would you expect to provide that support, and what would/did s/he do?

Comparison to other care:

How did the visit compare to an in-person visit? (IF VIDEO, How did the visit compare to a telephone visit with your provider?)
 Probe: perception of time

Do you see phone/video visits as more or less convenient compared to in-person visits? Why?

Was there anything different you did to prepare for this type of visit compared to an in-person visit?

If this was NOT your first phone/video visit experience, can you tell us how this compared to another visit on the phone/computer?

Conclusion

Did your feelings about having a phone/video visit change from when it was first offered to how it ended up?
